# Capnocytophaga canimorsus Meningitis Complicated by Septic Shock: The Use of Extracorporeal Blood Purification Techniques

**DOI:** 10.7759/cureus.59841

**Published:** 2024-05-07

**Authors:** Rosanna Carmela De Rosa, Fabrizio Falso, Gianfranco Viola, Massimiliano Barberio, Roberto Giurazza

**Affiliations:** 1 Department of Anesthesia and Intensive Care, AORN Ospedali dei Colli - "D. Cotugno" Hospital, Naples, ITA

**Keywords:** β-thalassemia major, polymyxin b hemoperfusion, acinetobacter baumannii, toraymyxin, oxiris, blood purification therapy, extracorporeal therapies, septic shock, meningitis, capnocytophaga canimorsus

## Abstract

*Capnocytophaga canimorsus* is a Gram-negative bacterium, commonly found as a commensal germ in the oral cavity of dogs and cats. It is an opportunistic pathogen, but, in specific situations, it can cause very severe diseases, including arthritis, pleuritis, endocarditis, sepsis, and, in extremely rare cases, meningoencephalitis. The predisposing situations include immunosuppression, liver cirrhosis, splenectomy, hemochromatosis, beta thalassemia major (Cooley’s anemia), and alcohol abuse. In this report, we describe the case of a 48-year-old male patient, with a medical history of several predisposing conditions, who developed a severe case of meningoencephalitis caused by *C. canimorsus*, following a dog bite on his hand. The patient was successfully treated for his meningitis, but subsequently he developed a hospital-acquired septic shock from *Acinetobacter baumannii*, which was treated with targeted antibiotic therapy and sequential extracorporeal blood purification therapies using Oxiris™ and Toraymyxin™ hemofilters.

## Introduction

The incidence of *Capnocytophaga canimorsus* invasive infection in the general population is 0.67 cases per million per year, but it is higher in immunocompromised hosts [1]. Meningoencephalitis by *C*. *canimorsus* is an extremely rare disease, occurring in particular conditions, with non-specific clinical presentation and negative Biofire™ Film Array Meningitis Encephalitis (FAME) test. Consequently, prompt diagnosis and appropriate antibiotic therapy are frequently delayed. Long hospital stays in these patients can be complicated by sepsis with antimicrobial resistance (AMR) [2]. In this setting, extracorporeal blood purification therapies can support systemic antibiotic therapy. Our case of rare meningoencephalitis by *C. canimorsus* shows the choice and efficacy of these extracorporeal techniques during septic complications.

## Case presentation

A 48-year-old male patient was admitted to the Emergency Department of our hospital with confusion, delirium, hyperpyrexia (39°C), vomiting, and rigor nucalis two days after a dog bite. Blood tests showed elevated white blood cells (WBC) (39,540 cells/μL with 84.1% of neutrophils), elevated C reactive protein (CRP) (132.16 mg/L), and elevated procalcitonin (PCT) (10.1 ng/mL). His medical history was complex: homozygous beta thalassemia major (Cooley’s anemia) treated with blood transfusions twice a month, hemochromatosis, thalassemic cardiomyopathy, liver cirrhosis caused by hemochromatosis and hepatitis C virus infection, previous splenectomy, extramedullary erythropoiesis, diabetes mellitus, hypothyroidism, hypogonadism, and previous seizures.

The suspicion of meningitis was so high that after brain computed tomography (CT) scan, a lumbar puncture was performed. Cerebrospinal fluid (CSF) examination showed turbidity, hypercellularity (1,739 WBC/μL with 92.5% polymorphonucleates), hypoglycorrhachia (2.10 mg/dL compared to 133 mg/dl of blood glucose), and hyperproteinorrachia (421.6 mg/dL). Biofire™ FAME test on CSF resulted negative. One specimen of CSF was sent to the microbiology laboratory for culture. Due to persistent agitation, on the same day, the patient was transferred to our intensive care unit (ICU), where he was sedated, intubated, and mechanically ventilated.

Upon admission, we performed cultures from multiple samples and started (1) a steroid therapy based on intravenous (IV) dexamethasone 0.15 mg/kg/die with subsequent slow tapering over one week and (2) an empiric broad-spectrum antibiotic therapy with IV meropenem 2 g thrice a day (tid) and linezolid 600 mg twice a day (bid). In the following days, brain CT and magnetic resonance imaging (MRI) scans showed signs of encephalitis. All the cultures performed were negative, except for the peripheral and central blood cultures showing positivity to *C. canimorsus*, susceptible to meropenem (Table 1).

**Table 1:** Capnocytophaga canimorsus antibiogram obtained from blood culture. MIC, minimum inhibitory concentration; S, susceptible; R, resistant; I, intermediate The above antibiogram was interpreted according to the EUCAST 2023 breakpoint tables

Tested antibiotic	MIC
Meropenem	0.08 (S)
Tigecycline	1 (R)
Amikacin	0.5 (S)
Amoxicillin/clavulanic acid	0.25 (S)
Cefepime	0.19 (S)
Cefotaxime	0.12 (S)
Ceftazidime	0.12 (S)
Ciprofloxacin	0.5 (I)
Piperacillin/tazobactam	0.5 (S)
Ceftazidime/avibactam	0.12 (S)
Ceftolozane/tazobactam	0.12 (S)

Based on the antibiograms of the blood cultures, meropenem and linezolid were stopped in accordance with carbapenem-sparing policies, and IV ceftriaxone 2 g bid was started. The outcome was positive until the extubation on day 7, when the patient showed good sensorium but hyposthenia in the four limbs, which our neurologist attributed to the meningoencephalitis, also according to the MRI performed. On day 11, our patient was transferred to the infectious diseases ward.

After only four days (on day 15), the patient returned to our ICU in septic shock, with hemodynamic instability requiring noradrenaline and vasopressin, hyperlactatemia, pharmaco-resistant hyperpyrexia (41°C), Glasgow Coma Scale (GCS) <9, elevated WBC, CRP, and PCT, normal endotoxin activity assay (EAA), and initial signs of acute kidney injury (AKI). He was sedated, intubated again, and mechanically ventilated. We started a continuous renal replacement therapy (CRRT) with the Oxiris™ (Baxter International, Deerfield, IL, USA) hemofilter for 72 hours, also using the extracorporeal circuit as a means of external physical cooling system. We also started an infusion of immunoglobulin M (IgM)-enriched human immunoglobulins (Pentaglobin™) (Biotest, Dreieich, Germany) 250 mg/kg over 12 hours daily for three days. When bronchoalveolar lavage (BAL) highlighted pandrug-resistant (PDR) *A*. *baumannii* (Table 2), which was absent in previous cultures, nebulized colistin 2,000,000 international units (IU) tid was added to IV meropenem 2 g tid and tigecycline 50 mg bid already ongoing from the discharging ward.

**Table 2:** Acinetobacter baumannii antibiogram obtained from bronchoalveolar lavage (BAL) culture. Bacterial count: 1,000,000 CFU/mL. CFU, colony forming unit; MIC, minimum inhibitory concentration; S, susceptible; R, resistant; IE, insufficient evidence The above antibiogram was interpreted according to the EUCAST 2023 breakpoint tables

Tested antibiotic	MIC
Amikacin	>32 (R)
Aztreonam	32 (R)
Ciprofloxacin	>8 (R)
Tigecycline	2 (IE)
Cefepime	32 (R)
Cefotaxime	>16 (R)
Ceftazidime	>32 (R)
Colistin	>32 (R)
Ertapenem	>1 (R)
Gentamycin	>8 (R)
Imipenem	32 (R)
Meropenem	>16 (R)
Piperacillin/tazobactam	>64 (IE)
Temocillin	>16 (R)
Trimethoprim/sulfamethoxazole	>8 (R)
Ceftolozane/tazobactam	>32 (R)
Ceftazidime/avibactam	>32 (R)
Cefiderocol	>256 (IE)

Afterward, the patient's general conditions improved with progressive weaning from vasopressors and with the normalization of CRP, PCT, and renal function despite persistence of fever and neurological impairment. Since on day 24 the EAA value was 0.9, we started another extracorporeal depuration technique: two cycles, lasting two hours each, of polymyxin B hemoperfusion (PMH) using the Toraymyxin™ (Toray Industries Ltd., Chuo-ku, Tokyo, Japan) hemofilter on days 24 and 25. After the first cycle of Toraymyxin™, the EAA value was 0.68, and after the second cycle, it was 0.49. In the following days, the EAA was constantly <0.5; PCT and CRP were in the normal range as well. On day 27, vital signs and sensorium were normal, and the patient passed a spontaneous breathing trial; thus, he was extubated. On day 29, we stopped the antibiotic therapy, except for nebulized colistin. The patient was transferred again to the previous ward on day 39, with negative BAL culture.

The patient gave written informed consent for the publication of this case report.

## Discussion

In our case report, we present two main issues: (1) a rare meningitis caused by *C. canimorsus* and (2) an *Acinetobacter baumannii* hospital-acquired pneumonia (HAP) with septic shock.

*Capnocytophaga canimorsus *is an opportunistic Gram-negative bacterium, commonly found in the oral cavity of dogs and transmitted to humans with bites and saliva [3]. In immunocompromised patients, *C. canimorsus* can cause septic shock, multiorgan failure, and, very rarely, meningoencephalitis [3]. Predisposing conditions include splenectomy, liver cirrhosis, alcohol abuse, hemochromatosis, beta-thalassemia major (Cooley’s anemia), and immunosuppression. As *C. canimorsus* has a polysaccharide capsule that gives it high resistance against the complement and macrophage-mediated phagocytosis and also needs high quantities of iron to grow efficiently, splenectomy and hemochromatosis are two main risk factors for systemic infection [4]. *Capnocytophaga canimorsus* can take up to 14 days to grow on blood cultures, its clinical presentation is often non-specific, and the Biofire™ FAME test - performed on CSF in case of meningitis and encephalitis - does not include it. The chances of survival depend on prompt diagnosis with blood cultures and appropriate antibiotic therapy [5]. Therefore, clinicians should strongly suspect *C. canimorsus *infection in patients with predisposing factors, especially after dog bites [6].

The second issue we present in this paper is HAP, caused by PDR *A. baumannii*, causing refractory septic shock and requiring sequential extracorporeal blood purification therapies. In accordance with surveillance cultures, our patient was not infected in the ICU, but he showed colonization and then developed pneumonia by *A. baumannii* during his stay in the ward.

In septic shock, the so-called “cytokine storm” and uncontrolled release of mediators cause organ dysfunctions. Different extracorporeal blood purification techniques aim to decrease their circulating levels, mitigating their detrimental systemic effects [7,8]. These techniques include different filters based on their cut-off, which is the smallest molecular weight of a solute that a filter can retain.

Malard et al. published an interesting study in 2018; using Oxiris™, Cytosorb™, and Toraymyxin™, they analyzed the removal rates of cytokines and endotoxin (i.e., lipopolysaccharide [LPS]) after in vitro hemoperfusion of human plasma, which was preincubated with pathologic quantities of inflammatory mediators. Oxiris™ was the only hemofilter able to significantly absorb both cytokines and LPS, with similar LPS removal to Toraymyxin™ and similar cytokine clearance to Cytosorb™. On the other hand, Toraymyxin™ showed only LPS adsorption, with no cytokine clearance, and Cytosorb™ showed only cytokine removal, with no LPS clearance. These different capacities could enable a more personalized and precision treatment in different clinical scenarios [9].

In 2022, Li et al. reviewed the use of Oxiris™ in septic patients, concluding that Oxiris™-CRRT was associated with reduced cytokine and endotoxin levels, and improved hemodynamics; however, due to the complexity of ICU patients, its effect on mortality rate and length of hospital stay remains inconclusive [10].

Our choice of Oxiris™ hemofilter was linked to initial AKI, normal value of EAA, cytokine storm, and refractory fever. It was used for 72 hours in continuous venovenous hemodiafiltration modality, with regional citrate anticoagulation and external physical cooling.

We used Toraymyxin™ hemoperfusion in a second moment, when the patient had elevated EAA (0.9) but normal renal function. After two cycles of two hours each with a 24-hour interval, a fall in EAA with subsequent stable values <0.5, resolution of fever, normalization of CRP and PCT, and significant improvement in the general clinical condition were observed.

The Toraymyxin™ hemofilter consists of polystyrene fibers with immobilized polymyxin B, a polycationic antibiotic that binds to the electronegative lipid A portion of the endotoxin, neutralizing its toxicity [11]. The EUPHAS trial was the first randomized controlled trial that examined the effects of PMH on 28-day mortality, enrolling patients with abdominal sepsis and septic shock due to Gram-negative bacteria. The authors showed a statistically significant reduction in Sequential Organ Failure Assessment (SOFA) scores, higher mean arterial pressure, and lower mortality in the PMH group [12]. The previous findings were confirmed by the EUPHAS2 study that identified a critical threshold of EAA as ≥0.6 [13].

## Conclusions

We present a rare case of meningoencephalitis caused by *C. canimorsus* in a patient with several predisposing conditions for invasive infection. Although very rare, this infection should always be kept in mind when dealing with patients who have recently been exposed to dog bites in the presence of risk factors. Since the clinical presentation is vague and *C. canimorsus* is not included in the Biofire™ FAME test, a high degree of suspicion is needed, as prompt diagnosis and appropriate therapy are fundamental for survival and avoidance of long-term neurological complications. Due to severe immunodepression, the aforementioned patient developed a PDR *A. baumannii *pneumonia, resulting in septic shock, treated with antibiotic therapy, as well as extracorporeal blood purification therapies, with Oxiris™ hemofilter first and Toraymyxin™ hemoadsorption afterward. We strongly emphasize that one treatment does not exclude another, but the use of different filters must be linked to blood circulating levels of EAA, sepsis mediators, and degree of AKI.
